# Application of CAD-CAM 3D Technology in Designing a Molar Distalization Device with Skeletal Anchorage: A Case Report

**DOI:** 10.3390/dj12120417

**Published:** 2024-12-18

**Authors:** Martina Mezio, Alessandra Putrino, Ersilia Barbato, Stefano Pandolfi, Michele Cassetta

**Affiliations:** 1 Department of Oral and Maxillofacial Sciences, School of Dentistry, Sapienza University of Rome, 00161 Rome, Italy; alessandra.putrino@uniroma1.it (A.P.); ersilia.barbato@uniroma1.it (E.B.); michele.cassetta@uniroma1.it (M.C.); 2 Private Practice, 00100 Rome, Italy; ortodonticastefano@gmail.com

**Keywords:** mandibular molar, distalization, customized appliances, orthodontics, tooth movement, computer guided surgery, surgical guide, skeletal anchorage, miniscrew, CAD-CAM technology

## Abstract

**Objectives:** Mandibular molar distalization is a complex orthodontic movement due to anatomic and biomechanical limitations. The opportunity to use a custom-made appliance with skeletal anchorage should be an advantageous alternative to traditional solutions: multiple extractions, interproximal reductions, vestibular inclination of incisal group. **Methods**: A 14-year-old female patient with Class II malocclusion and ectopic upper and lower canines was treated in the lower arch with a custom-made appliance anchored on a mini-screw in the right buccal-shelf where the ectopy and crowding was severe. The miniscrew was connected to a rigid arm with a rail equipped with a coil that activated promoted the distalization of first and second molars bonded with metallic bands. **Results**: After 8 months of treatment with activations repeated every 4 weeks, an effective distalization has been reached. **Conclusions**: Mandibular molars’ distalization is a challenging orthodontic result to achieve. When the need to obtain space cannot be beneficially obtained with conventional approaches, and distalization of the lower molars could be desirable, a custom distalization device with skeletal anchorage and biomechanics based on a pressed coil sliding on a rigid arm is an efficient solution.

## 1. Introduction

Mandibular molar distalization can be considered a valuable orthodontic technique for correcting malocclusions without the need for extractions, particularly in cases of dental crowding. Upper molar distalization can be utilized to correct Class II malocclusions, while lower molar distalization is employed in the treatment of Class III malocclusions. Additionally, simultaneous distalization of both upper and lower molars may be used to correct maxillary and mandibular prognathism [1]. Historically, most research and clinical practices have focused on maxillary molar distalization, while the distalization of mandibular molars remains less explored due to the anatomical and biomechanical complexities associated with the denser mandibular bone and the limited space posteriorly [2]. The distal movement of mandibular molars can be achieved using various methods, including clear aligners, temporary anchorage devices (TADs), micro-implants, and miniplates, each with distinct biomechanical implications and clinical outcomes [3,4,5]. Recent reviews delve into the various techniques used for mandibular molar distalization, evaluating their efficacy, associated challenges, and the impact on adjacent dental and soft tissues. They focus on clear aligners and skeletal anchorage methods, highlighting their applications, outcomes, and limitations in the distalization of mandibular molars [5,6]. The aim of this case report is to document a challenging and effective method to obtain a stable lower molar distalization using a custom-made appliance with a rail and a rigid arm connected to a skeletal anchorage obtained through a mini screw inserted in the buccal shelf.

## 2. Case Report

A case of a female 14-year-old patient in permanent dentition treated for orthodontic management of Class II molar and canine relationship with skeletal anchored custom-made appliance is reported. The clinical case description focuses specifically on the distalization of the right mandibular molars. An upper distalization was also performed with a custom-made device.

### 2.1. Diagnosis and Etiology

In January 2024, the 14-year-old patient presented for orthodontic evaluation at the Orthodontic Unit of the Oral and Maxillo-Facial Sciences Department, Sapienza University of Rome, Italy. The patient was in good general health and the parents reported no history of previous illness or surgeries.

**Figure 1:**
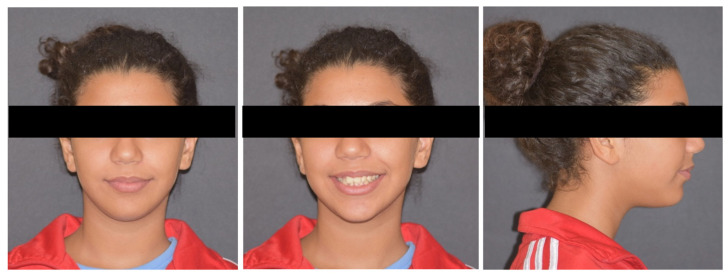
Pre-treatment extraoral photographs.

The extra-oral examination revealed an ovoid facial shape with an acceptable symmetry. The lips were competent, and the facial profile was convex (Figure 1).

**Figure 2:**
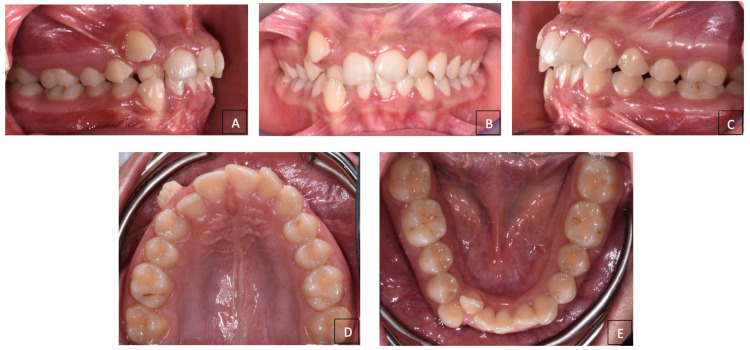
Pre-treatment intraoral photographs. (**A**) Right lateral intraoral view; (**B**) frontal intraoral view; (**C**) left lateral intraoral view; (**D**) upper occlusal intraoral view; (**E**) lower occlusal intraoral view.

The intra-oral examination established that the patient was in permanent dentition up to the second molars of both arches and had a Class II molar and canine relationship not evaluable. The patient presented a severe upper and lower crowding, along with vestibular ectopy of the upper and lower canines and lingual position of right lower lateral incisor (Figure 2A–E).

**Figure 3:**
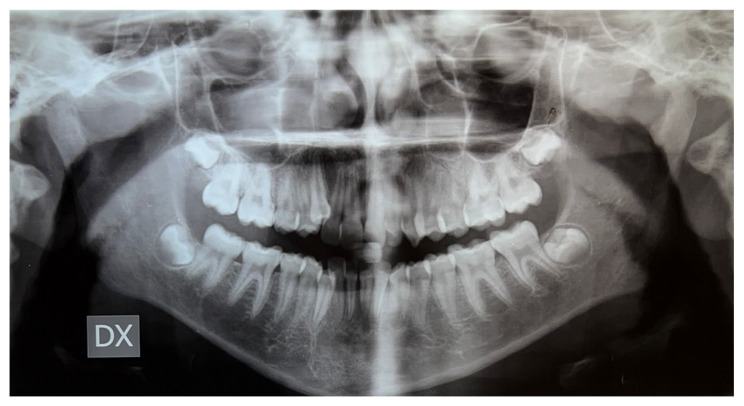
Pre-treatment orthopantomography.

Having collected the photographic and radiographic documentation, different possible therapeutic paths were evaluated. The pre-treatment orthopantomogram is illustrated in Figure 3.

Once the cephalometric analysis was performed using the Oris Ceph software Rx CE (Oris Ceph, Elit Computer 8.3.1, Vimodrone, Milan, Italy), it revealed the following dentofacial characteristics: Class II skeletal malocclusion due to protrusion of the maxilla (SNA 86.1°, SNB 80.4°, ANB 5.7°), hyperdivergence (FMA 29.8°), and increased inclination of incisors (IMPA 98.9°, FMIA 51.3°). Additionally, the following parameters were assessed for the evaluation of the proportions and relationships between the different facial structures: facial height Y = 120.5 mm; SnGoMe = 37.3°; SNPg = 80.5°; NgoMe = 81°; NL/ML = 31.7°; NSL/NL = 4.4°; NSL/ML = 36.0°.

### 2.2. Treatment Objectives

The primary objective was the management and correction of the Class II malocclusion and the correction of dentoalveolar alteration.

### 2.3. Treatment Alternatives

Both the dental and skeletal malocclusion can be managed through different alternative therapeutic approaches.

**Figure 4:**
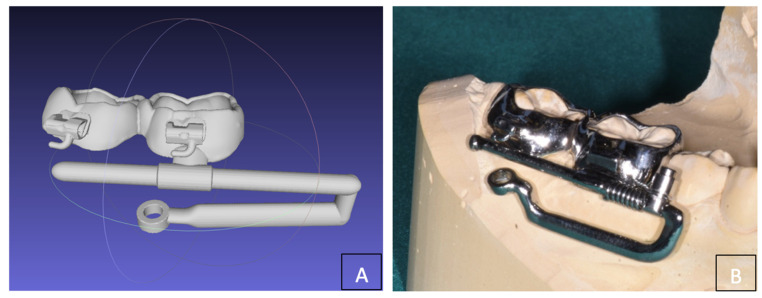
(**A**) Planning of the customized skeletally anchored device; (**B**) customized distalization device (Ortodontica Italia, Rome, Italy).

**Figure 5:**
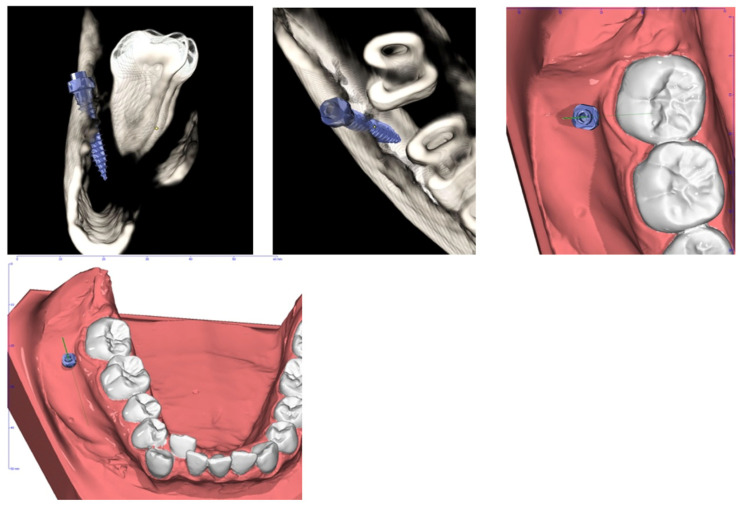
Planning of miniscrew insertion (Ortodontica Italia, Rome, Italy) on CBCT (Cone Beam Computed Tomography) from different views.

The primary therapeutic option would involve an extractive orthodontic approach, which includes the extraction of the four first premolars. This aims to resolve crowding and malocclusion by distalizing the posterior segments of both arches. A second option could involve the extraction of the upper canines and of the right lower canine, leading to a resolution of the crowding and malocclusion. Given the positive experience documented in the literature [6], we evaluated the application of a Distal Jet device on miniscrews for the upper arch. For the lower arch a customized device (Figure 4A,B) was designed consisting of two bands on 4.6 and 4.7 teeth with a vestibular “rail” adjacent to the gingival margin (parallel to the occlusal plane) with a rigid arm for connection to the miniscrew (BENEfit, PSM medical solutions, Tuttlingen, Germany) inserted in the buccal shelf (Figure 5). In agreement with the subject’s guardians, the second option was planned, and the treatment was preceded by the extraction of the lower right third molar (4.8).

A non-extraction therapeutic approach was also chosen for the upper arch, using a Distal Jet with skeletal anchorage to achieve bilateral molar distalization. In this case, no extractions of the upper third molars were performed.

### 2.4. Treatment Progress

**Figure 6:**
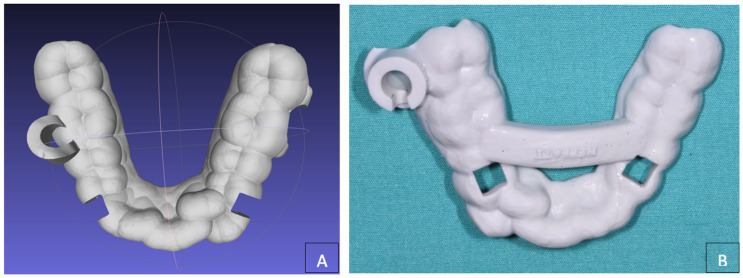
(**A**) Planning of the Customized surgical template; (**B**) customized surgical template.

The entire procedure and risk of failure’s distalization associated with miniscrew loss and custom-made appliance were explained to the patient and her parents. Signed informed consent for the publication of the clinical case, including images and anonymized data, was obtained. The patient was asked to carry out a CBCT, and digital dental models were created from intraoral scanning. The DICOM files of the CBCT and the stl file of the scan allowed us to design a surgical guide to improve the safety and accuracy of the miniscrews insertion. The TADmatch 3D module of the Onyxceph3™ software (Image Instruments, Chemnitz, Germany) was used for this purpose. The surgical guide was fabricated using the TruPrint 1000 (TRUMPF Homberger S.r.l, Buccinasco, Italy) (Figure 6A,B), and the mandibular distalizing appliance was manufactured using laser melting technology with cobalt chrome metal powder (Stratasys OrhoDesktop; Stratasys, Rehovot, Israel).

The self-drilling titanium miniscrews used (BENEfit, PSM medical solutions, Tuttlingen, Germany) were chosen for their length and diameter during the planning phase, based on the anatomical features of the palate and mandible bone. In the lower arch, a miniscrew (length: 13 mm; diameter: 2.3 mm) was inserted into the buccal shelf on the right side of the mandible. It was connected to a rigid steel alloy arm and a rail adjacent to the gingival margin of the first and second right molars, which were covered with cemented metallic bands. The same procedure was applied in the upper arch, where two miniscrews (length: 11 mm; diameter: 2.3 mm) were inserted into the palatal vault in a paramedian position using a surgical guide.

#### 2.4.1. Surgery

**Figure 7:**
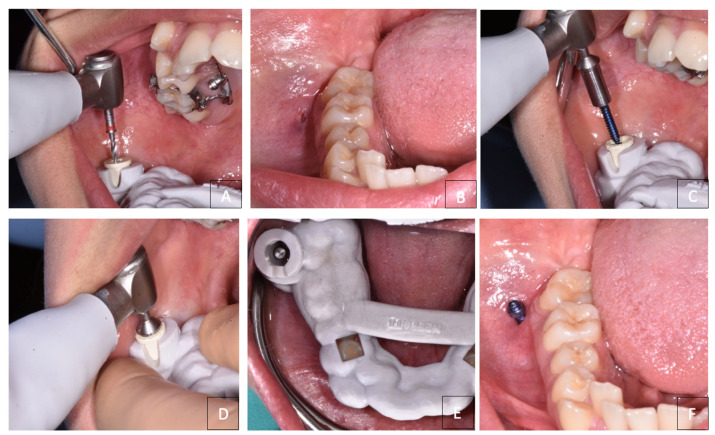
(**A**,**B**) Pre-drilling to guide miniscrew insertion; (**C**,**D**) miniscrew insertion; (**E**) intraoral occlusal view of surgical guide insertion; (**F**) miniscrew in place.

**Figure 8:**
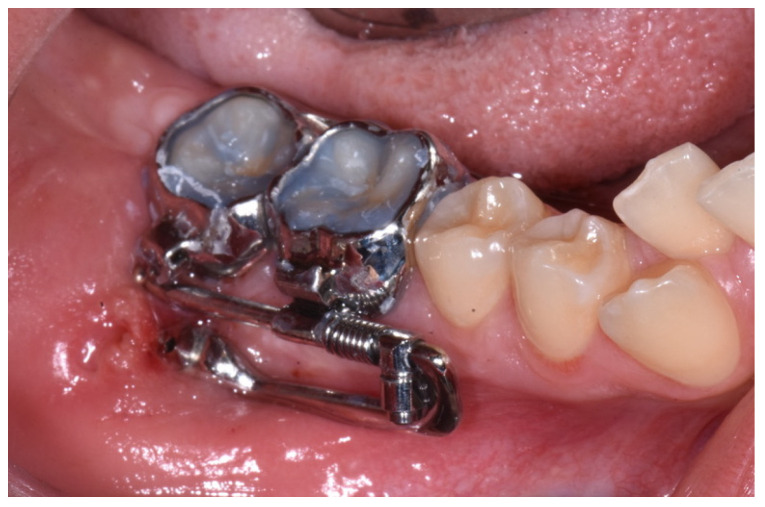
Intraoral application of the orthodontic device.

On the day of the planned surgery, the patient rinsed her mouth with a 0.2% chlorhexidine solution before the insertion of the miniscrew. Then, a 2% carbocaine anesthetic solution with adrenaline in the ratio 1:100,000 was locally infiltered. The stability and congruity of the surgical guide were checked, then, due to the hardness of the mandibular cortical bone a pre-drilling step was performed. Subsequently, the miniscrew was inserted using a torque-controlled contra-angle handpiece. During the pre-drilling phase, the drill was in contact with the bone and was used at 550 rpm under water irrigation. The miniscrew insertion torque was set at a speed of 25 rpm and 40 N cm. The desired depth of insertion was checked with a stop on the screw holder, which was perfectly adapted to the master tube in the guide hole of the surgical guide. After the miniscrew insertion, primary stability was evaluated in relation to the insertion torque, before connecting the custom-made distalizing appliance (Figure 7A–F). A fixation screw system was then used to connect the miniscrew with the custom-made appliance. Molar bands were cemented to the molars (Figure 8).

The same procedure was performed on the upper arch; however, pre-drilling was not necessary due to the reduced thickness of the palatal cortical bone. After evaluating the primary stability of the palatal miniscrew, the Distal Jet orthodontic device was applied.

#### 2.4.2. Orthodontic Activation

The devices were activated after application, with activations repeated every four weeks. Efficient activation of both devices required full pressure from the Ni-Ti helical springs inserted into the rails. The force released by the springs was approximately 250 N.

### 2.5. Treatment Results

**Figure 9:**
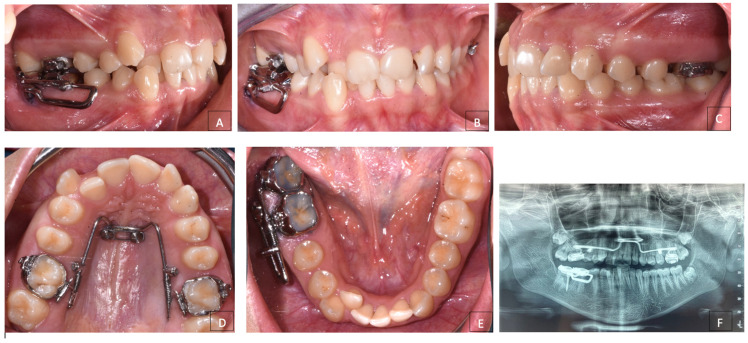
Post-treatment intraoral photographs. (**A**) Right lateral intraoral view; (**B**) frontal intraoral view; (**C**) left lateral intraoral view; (**D**) upper occlusal intraoral view; (**E**) lower occlusal intraoral view; (**F**) Post-treatment orthopantomography.

After 8 months, effective molar distalization was achieved in both the mandibular and maxillary arches, and a control orthopantomogram was performed to assess the integrity of the roots, evaluate potential root resorption, and check for periodontal pathology. None of these complications were observed (Figure 9A–F). Additionally, due to the distalization, the necessary space was created to align all teeth, and the correct occlusal relationships were obtained. The orthodontic treatment will be finalized with multibracket orthodontic therapy. Both devices will remain passive throughout the duration of the fixed therapy to maximize anchorage, prevent molar mesialization, and maintain molar relationship. The stability of the miniscrews was monitored monthly using percussion testing, which confirmed the continued effectiveness of the distalization device throughout the entire treatment [7,8]. The treatment produced positive results, without evident complications related to the soft tissue response or loss of anterior anchorage, demonstrating good control and effective management of undesirable dento-alveolar effects.

To date, the distalization of mandibular molars remains one of the most complex outcomes to achieve, primarily due to anatomical limitations and the challenge of finding adequate posterior anchoring points. When space recovery cannot be managed through IPR or compensation via the vestibular inclination of the incisal group, extractions may appear to be the only viable strategy.

## 3. Discussion

Molar distalization has always been one of the most complex challenges for orthodontists, primarily due to the difficulties in ensuring effective anchorage and controlling molar movement without compromising occlusal stability and periodontal health. The therapeutic alternatives in this case report would have been the extraction of the first premolars or the canines. Both options should be followed by a long and complex therapeutic phase using multibracket orthodontic therapy. Although the extraction-based approach can be an effective solution for many cases of dental malocclusion and crowding, the scientific literature highlights several potential drawbacks, including aesthetic and functional complications, skeletal changes, the need for more complex orthodontic biomechanics, periodontal issues (such as gingival recession, loss of periodontal attachment, and root resorption), a higher risk of relapse, longer treatment times, and psychological challenges [9,10,11,12,13]. For these reasons, it is essential for the orthodontist to perform a thorough, personalized assessment to determine whether an extraction approach is truly necessary or if alternative non-extraction options might be more appropriate for the patient. To address these drawbacks, another alternative to the extraction approach is the possibility of attempting the distalization of both arches using skeletally anchored molar distalization devices.

The introduction of skeletal anchorage in orthodontics has opened numerous therapeutic possibilities and sparked significant academic interest in this field [14].

This case report presents an innovative technique for molar distalization using skeletal anchorage, successfully applied to both the upper and lower arches with customized orthodontic devices. The use of skeletal anchorage, combined with the tailored design of the devices, resulted in optimal outcomes, overcoming the limitations of traditional techniques and reducing the risk of side effects such as extrusion or mesialization of the molars [6]. The use of skeletal anchorage devices for molar distalization, in addition to eliminating the need for extractions, simplifies patient compliance by limiting it to maintaining good oral hygiene. This approach significantly reduces the treatment time for subsequent fixed multibracket therapy and minimizes the need for patient intervention, such as the use of intraoral elastics [15].

The prognosis of molar distalization with skeletal anchorage in a hyperdivergent patient with dental crowding is highly dependent on the ability to control vertical dimension, prevent molar extrusion, ensure stable anchorage, and monitor periodontal and root health. It is also essential to maintain the stability of the miniscrews throughout the treatment. The prognosis will be favorable if these factors are properly managed through the use of appropriate orthodontic biomechanics, precise planning of mini-implant placement, and an optimized design of orthodontic devices with controlled force vectors.

In hyperdivergent patients, one of the main challenges during molar distalization is controlling unwanted vertical movements and the inclination of the incisors. Skeletal anchorage is particularly beneficial in these cases, as traditional anchorage methods (such as anchoring on premolars or molars) may not adequately prevent molar extrusion or loss of anterior anchorage. A key factor in successful molar distalization is the orientation of the force vector, which must be carefully controlled to avoid increasing the vertical dimension. To achieve distalization without extrusion, the force vector should be predominantly horizontal [16,17,18].

In this case report, the decision to place the miniscrew in the buccal shelf and the design of the orthodontic device were specifically intended to create a horizontal force vector as parallel as possible to the occlusal plane. The goal was to achieve bodily distalization of the molars without causing undesirable vertical effects. The lower distalization device was designed such that both the vestibular ‘rail’, adjacent to the gingival margin, and the rigid arm connecting to the miniscrew were parallel to the occlusal plane, generating a purely horizontal force vector. For the upper arch, a skeletal anchorage Distal Jet was chosen, supported by two palatal miniscrews, which, according to the literature [6], does not cause vertical changes or molar extrusion. Therefore, the results of this case report demonstrate effective molar distalization in both the upper and lower arches, without compromising the vertical dimension.

The selection of the buccal shelf as the site for inserting skeletal anchorage devices offers several advantages. This location provides optimal bone density, ensuring greater stability for the mini-implants and significantly lower failure rates compared to those placed in the interradicular area. Furthermore, inserting miniscrews in the buccal shelf reduces the risk of damage to adjacent anatomical structures, including vascular structures, nerves, and dental roots. From a biomechanical standpoint, the ability to insert the mini-screws parallel to the long axes of the molar roots enables the application of direct and controlled forces, improving the direction of molar movement and the extent of molar distalization, without the need for repositioning the miniscrew [19,20,21].

An important aspect to consider during molar distalization treatment is the management of third molars. In this case report, only the right mandibular third molar (4.8) was extracted. Although the extraction of maxillary third molars may seem like a reasonable solution before applying the distalization device, the results of Altieri et al. [6] suggest that the presence of third molars does not significantly interfere with the distalization process, if they are not fully erupted, especially when skeletal anchorage devices are used. The extraction of the right mandibular third molar (4.8) was performed to facilitate molar distalization in the lower arch, as this process is generally more complex in the mandible due to its greater bone density and thicker cortical bone. In this case, the presence of the third molar could have hindered the desired movement and compromised the stability of the miniscrew.

Recent advances in mandibular molar distalization techniques showed that clear aligners can effectively induce controlled tooth movements, including distal tipping and bodily movement of molars. A study by Wu et al. [2] demonstrated that clear aligners effectively distalize mandibular molars, with significant differences observed between the initial and post-treatment stages. The study highlighted that the distalization rate of crowns was higher than that of roots, with second molars showing greater displacement than first molars, indicating that clear aligners are particularly efficient in moving the crown of the second molar compared to the first molar. This trend has been also observed by Putrino et al. who reported that the unpredictability of the effective tooth movement rate for the mandibular molars can also raise medico-legal issues [4]. However, clear aligner therapy for mandibular molar distalization may result in minor alterations in the position of the mandibular incisors and soft tissues, such as a slight increase in lower lip thickness and length, which could affect overall treatment aesthetics [2,6]. Despite these changes, clear aligners remain a non-invasive and relatively predictable option for mandibular molar distalization, especially when paired with digital treatment planning and evaluation tools like cone beam CT (CBCT) and cephalometric software [2,5,6].

Temporary anchorage devices (TADs) have been extensively used to provide absolute anchorage in complex orthodontic treatments [22]. Their use, combined with CAD-CAM techniques for the creation of custom devices, can be applied not only in cases of distalization but also in cases of tooth inclusion in the mandible. [23] These devices help circumvent the reciprocal forces that typically hinder distal tooth movement, thus enhancing the efficacy of the distalization process. According to Kang et al. [24], micro-implants placed between the roots of mandibular molars can significantly enhance distalization efficiency by providing a stable anchorage point that minimizes unwanted mesial movements of adjacent teeth. The study’s finite element analysis revealed that with micro-implant anchorage, the overall displacement of the first molar was maximized when starting positions of the molars were adjusted distally [24].

TADs have shown efficacy in achieving more pronounced distalization without requiring patient compliance, as seen in studies comparing different skeletal anchorage techniques. Yeon et al. [25] reported that the use of ramal plates and miniscrews resulted in varying degrees of distal molar movement, with ramal plates showing more significant distalization at both the crown and root levels compared to buccal miniscrews. The selection of anchorage type thus depends on the clinical scenario, with ramal plates being more suitable for cases requiring extensive distalization [25].

Beyond clear aligners and skeletal anchorage, several other approaches have been explored. For instance, the use of miniplates as an anchorage device has been effective, particularly in challenging cases involving adults, where bone density and lack of space complicate distalization. Miniplates provide robust anchorage that allows for significant distalization of molars without the typical side effects seen with other devices [26]. The finite element study by Zhu et al. [27] explored various configurations of molar movement, demonstrating that distalization combined with extrusion of premolars and molars could influence the overall stability and alignment of anterior teeth. This study highlighted the need to carefully consider periodontal support when designing distalization protocols to avoid complications like gingival recession or dehiscence.

Distalization of mandibular molars involves complex biomechanical challenges due to the higher bone density and limited space within the mandibular arch. Effective distalization requires careful planning of force vectors to prevent adverse effects such as mesial tipping of adjacent teeth or unintended extrusion of molars. Finite element studies have shown that clear aligners, when combined with skeletal anchorage like micro-implants, can improve the predictability and efficiency of molar distalization while minimizing unwanted side effects [24]. Moreover, the position and type of skeletal anchorage significantly influence the outcome of mandibular molar distalization. For example, using micro-implants in conjunction with different molar starting positions can optimize force distribution, enhancing the distal movement while preserving the overall dental arch integrity [24]. Additionally, the use of TADs allows for greater control over distal movement without requiring patient cooperation, which is crucial for achieving consistent results. Comparisons between TADs and other techniques, such as Class III elastics, have shown that TADs offer better control of molar movement with less undesired tipping or extrusion. Studies indicate that Class III elastics tend to cause distal tipping and extrusive forces on molars, whereas TADs promote bodily movement with minimal vertical displacement, making them preferable in high-angle cases where maintaining occlusal stability is critical [28].

Moreover, the use of ramal plates has been compared to buccal shelf miniscrews, with the former showing superior results in terms of distal movement and reduced vertical changes. This suggests that the choice of skeletal anchorage should be carefully tailored to the patient’s vertical growth pattern and specific malocclusion characteristics [29]. Mandibular molar distalization is increasingly recognized as a viable alternative to extraction-based orthodontic treatments, offering aesthetic and functional benefits also using appliances used for simultaneous maxillary molar mesialization and distalization in patients with a maxillary asymmetrical relationship [30]. Clear aligners and skeletal anchorage devices, such as TADs and micro-implants, are at the forefront of this evolution, providing clinicians with versatile tools to manage complex malocclusions. However, individual patient anatomy, the extent of distalization required, and the presence of third molars are critical factors influencing treatment planning and outcomes [25].

The skeletal anchorage molar distalization device represents a valid therapeutic alternative in cases where it is necessary to distalize the lower molars to resolve dental crowding and achieve a Class I molar relationship. This approach can be considered an alternative solution to extractions. To optimize the effectiveness of the device, it is recommended to extract the third molars, if present, to reduce occlusal interference and allow for more effective molar distalization. In some cases, molar distalization may not be sufficient to fully resolve the crowding issue or achieve the desired molar relationship, particularly in the presence of severe malocclusions or associated skeletal problems, which may require more complex treatment approaches. The application of the device involves the placement of a miniscrew in the buccal shelf of the mandible. Computer-guided miniscrew insertion is an advanced technique that ensures optimal precision and accuracy in its placement. This approach significantly reduces the risk of damaging surrounding anatomical structures, such as nerves and blood vessels, thereby improving the safety of the procedure. Furthermore, CAD-CAM technology enables the creation of a custom-made device, optimizing the distribution and direction of forces and improving treatment outcomes. To achieve optimal results, it is crucial to ensure the stability of the miniscrew. Therefore, the treatment is contraindicated in patients with altered mandibular anatomy, reduced bone density, uncontrolled periodontal disease, or poor oral hygiene. In these cases, the risk of complications, such as lack of primary stability or peri-implantitis (inflammation around the miniscrew), increases significantly, compromising the overall effectiveness of the treatment.

Future research should aim to refine the biomechanics of mandibular molar distalization, exploring new materials and anchorage systems that could enhance treatment predictability and reduce potential side effects. Additionally, long-term studies on the stability of distalized molars and the implications on mandibular arch integrity are essential to validate these techniques as mainstream alternatives to traditional orthodontic methods.

## 4. Conclusions

The use of a custom-made appliance with skeletal anchorage for effective distalization of the mandibular molars should be regarded as a valuable option when traditional extractive or compensatory approaches are not the best solutions. Clinical experience, supported by dental technologies such as CAD-CAM for surgical planning and the customization of properly designed devices, combined with 3D printing of a surgical guide to enhance miniscrew insertion, constitutes a successful strategy.

## Data Availability

No new data were created or analyzed in this study. Data sharing is not applicable to this article.
